# Vincamine induces cytoprotective autophagy via regulation of ampk/mtor signaling pathway in gentamicin-induced hepatotoxicity and nephrotoxicity in rats

**DOI:** 10.1038/s41598-025-06355-5

**Published:** 2025-06-20

**Authors:** Alaa Abouelhamd, Nourhan Elpry Mahmoud Shehata, Sara Mohamed Naguib Abdel-Hafez, Dalia H. Abu-Baih

**Affiliations:** 1 Department of Biochemistry & Molecular Biology, Faculty of Pharmacy, Deraya University, New Minia City, 61111 Minia Egypt; 2 Deraya Center for scientific Research, Deraya University, New Minia City, 61111 Minia Egypt; 3 Department of Histology and Cell Biology, Faculty of Medicine, Minia University, Minia, 61519 Egypt

**Keywords:** Vincamine, Gentamicin, Nephrotoxicity, Hepatotoxicity, Autophagy., Biochemistry, Molecular biology

## Abstract

Gentamicin (GET), a widely utilized aminoglycoside antibiotic for severe bacterial infections, is associated with significant hepatorenal toxicity. These adverse effects are frequently exacerbated by GET-induced oxidative stress and inflammation. This study aimed to evaluate the potential protective efficacy of vincamine (VIN) against GET-induced hepatic and renal damage. 4 groups of adult male rats were assigned: normal control (received CMC), GET (100 mg/kg, i.p.), VIN (40 mg/kg, p.o.), and GET/VIN (received both VIN and GET) for 7 days. Liver and kidney function tests were performed. Serum total antioxidant capacity (TAC) and tissue malondialdehyde (MDA) were quantified. To assess apoptosis, *Bax* and *Bcl-2* mRNA levels were quantified using real-time polymerase chain reaction (RT-PCR), while cleaved caspase-3 protein levels were measured using ELISA. Histopathological alterations were also examined. The implication of autophagy was assessed by detecting AMPK, beclin-1, LC3 and mTOR proteins. Our results indicated that VIN significantly attenuated GET-induced hepatotoxicity and nephrotoxicity by mitigating oxidative stress and apoptosis. Mechanistically, VIN modulated apoptotic pathways by upregulating the anti-apoptotic *Bcl-2* gene and downregulating the pro-apoptotic *Bax* gene. Notably, VIN potently enhanced autophagy through modulation of the AMPK/mTOR signaling pathway, evidenced by the upregulation of beclin1 and LC3 levels. Histopathological analysis further corroborated these findings, demonstrating that VIN markedly reduced the tissue damage associated with GET administration. VIN demonstrates potential as a cytoprotective agent against GET-induced hepatorenal toxicity. The protective effect of VIN may be attributed to its capacity to modulate the Bax/Bcl-2/Caspase-3-dependent apoptotic pathway and the AMPK/mTOR-mediated autophagy pathway.

## Introduction

Aminoglycosides (AGs) are a vital class of antibiotics used to combat a spectrum of bacterial strains, notably aerobic gram-negative bacilli infections^1^. These potent antimicrobials exert their bactericidal effects by disrupting the outer membrane of pathogenic bacteria, thereby increasing membrane permeability. This disruption facilitates the rapid influx of AG molecules into the bacterial cytoplasm, ultimately impeding protein synthesis and leading to cellular demise. Among the AGs, Gentamicin (GET) is a frequently employed in clinical settings to treat serious bacterial infections, including gram-positive and gram-negative^2^. Despite its robust antibacterial activity, the therapeutic utility of GET is curtailed by its propensity to induce nephrotoxic and hepatotoxic effects, posing significant challenges in treatment regimens^3,4^. The precise mechanisms underpinning the hepatorenal toxicity manifestations induced by GET remain elusive despite recent investigations shedding light on this enigma^5^.

Prior research has elucidated that the nephrotoxic effects of GET are intricately tied to its accumulation within tubular epithelial cells, particularly in the proximal tubules, and throughout the distal and collecting ducts. Upon exposure to GET, various pathogenic pathways are set in motion within these tubular cells, including oxidative stress, inflammatory responses, mitochondrial dysfunction, endoplasmic reticulum (ER) stress, necrosis, and apoptosis. These cellular events can culminate in cell death, impaired tubular function, and a decline in the glomerular filtration rate (GFR)^6^. Notably, oxidative stress emerges as a pivotal molecular mechanism driving the onset of GET-induced nephrotoxicity. Mitochondria-mediated generation of reactive oxygen species (ROS) is markedly augmented by GET, leading to detrimental impacts on nucleic acids, proteins, and lipids, thereby exacerbating cellular damage and dysfunction^7–9^.

The liver, a vital hub in the detoxification cascade, serves as a frontline defender against the continual onslaught of drugs and toxins. Extensive investigations in animal models have explored the deleterious impact of GET on hepatic function, revealing a complex interplay of numerous factors^10,11^. While the pathogenesis of GET-induced hepatotoxicity has been somewhat delineated, the precise sequence of events remains elusive^12^. Among the proposed culprits, oxidative stress and inflammation emerge as prime instigators of liver damage following GET exposure^5^. At the mechanistic level, AGs interact with iron to form a potent aminoglycoside-iron complex, which reacts with free radicals generated during the metabolism of arachidonic acid. This intricate interaction triggers a cascade of cellular responses, including apoptosis and inflammation^4,9,13^.

Autophagy, a sophisticated cellular defense mechanism, orchestrates the selective removal of dysfunctional mitochondria and misfolded proteins via the intricate autophagosome-lysosome pathway^14,15^. During cellular stress, autophagy emerges as a guardian of cell survival, thwarting apoptotic pathways and preserving cellular homeostasis^16,17^. While autophagy typically operates within defined boundaries under normal physiological conditions, strong inducers have been found to significantly disrupt autophagic responses such as ischemia/hypoxia, infection, and fasting. The dynamic process of autophagic flux unfolds in a coordinated sequence, beginning with phagophore formation and the targeted sequestration of damaged organelles^18^. Consequently, autophagy activation plays a critical role in mitigating the toxicity induced by GET, shedding light on its therapeutic potential in countering GET’s adverse effects^19,20^.

It is noteworthy to emphasize that beclin-1 serves as a robust marker of autophagic dynamics. It forms autophagosomes, which are essential for the process of autophagic sequestration. Consequently, a decline in beclin-1 levels is indicative of compromised autophagic activity^21,22^. Notably, the regulation of autophagic processes is intricately linked to the activation of AMP-activated protein kinase (AMPK) and the modulation of the mammalian target of rapamycin (mTOR) pathway^22,23^. Intriguingly, previous investigations have unveiled that the activation of the AMPK/mTOR pathway can offer partial alleviation of GET-induced toxicity in rodent models, suggesting the therapeutic potential of targeting these pathways to ameliorate cellular damage^24^.

Maintaining tissue homeostasis hinges on the delicate orchestration of autophagy and apoptosis, critical processes that govern cellular fate^16,17^. Autophagy, a mechanism that enhances cellular resilience, promotes cell viability by clearing cellular debris and supporting cellular health. In contrast, apoptosis is a process that leads to programmed cell death, selectively removing severely compromised cells to maintain tissue integrity^14,17^. In many disorders, the intricate relationship between autophagy and apoptosis has been thoroughly studied^25,26^. Autophagy has demonstrated its ability to curb apoptotic cell death, particularly in experimental settings involving neurological disorders^27^ and liver injuries^28^.

Despite its pivotal role in regulating various aspects of normal kidney function and kidney-related diseases, such as renal development, aging, and tubular injuries, the role of autophagy in the kidney is still being investigated^29^. The precise alterations in the process of autophagy within the pathogenesis of renal diseases, and the subsequent impact of these alterations on renal function, remain unclear^30^. Recent findings suggest that the functionality of autophagy plays a pivotal role in the development of liver pathologies, including non-alcoholic fatty liver disease (NAFLD) and alcoholic fatty liver disease (AFLD), further exacerbating hepatotoxicity induced by viral infections, aflatoxins, or heavy metals^31^. Moreover, the recent scrutiny of the role of autophagy in liver disease, including hepatocellular carcinoma (HCC), sheds light on its intricate involvement in disease modulation and progression within hepatic pathologies^32–34^.

For millennia, medicinal plants have been pivotal in the management of human ailments, providing a rich source for the development of numerous pharmaceutical agents. Vincamine (VIN), a monoterpenoid indole alkaloid extracted from *Vinca minor* (lesser periwinkle) leaves, has been extensively researched since the 1950s, revealing a diverse array of medicinal properties such as antioxidant, anti-apoptotic, hepatoprotective, nephroprotective, and neuroprotective effects^35,36^. This compound has shown promise in mitigating cisplatin and methotrexate-induced nephrotoxicity through its anti-inflammatory and antioxidant properties^37,38^. Additionally, VIN has demonstrated effectiveness in alleviating tamoxifen-induced hepatic cell damage by reducing oxidative stress, apoptosis, and inflammatory responses^39^. Despite these findings, studies on VIN’s protective effects against GET-induced hepatorenal toxicity and its relation to autophagy are rare. Therefore, this study presents an innovative approach to understanding the protective effects of VIN against GET-induced hepatorenal damage through the induction of autophagy.

## Materials and methods

### Drugs and chemicals

GET was obtained from Memphis (Garamycin^®^ ampule; Memphis, Egypt). VIN was a gift from Hochster Pharmaceutical Industries, Cairo, Egypt.

### Experimental animals

The investigations were conducted in accordance with the National Institutes of Health Guide for the Care and Use of Animals. and received approval from the Ethics Committee at Deraya Center for Scientific Research, Deraya University, Minia, Egypt (Ethical approval no DCSR-03024-10). The study is reported in accordance with ARRIVE guidelines^40^. 24 male wistar rats weighing between 220 and 250 g were acquired from the Animal house at Deraya Center or Scientific Research, Deraya University. The animals were housed in separate cages with twelve-hour light/dark cycles and were given access to fresh water and food.

### Experimental design

Following a one-week accommodation, rats were assigned to four groups, with six rats in each group and all received treatment for 7 days:

1. Group I (normal control): given the vehicle (0.5% carboxymethyl cellulose, (CMC) orally once a day.
2. Group II (VIN): rats received VIN (40 mg/dl) in 0.5% CMC^37,38^, administered orally via gavage.
3. Group III (GET): rats received GET (100 mg/kg /day, i.p.)^41,42^.
4. Group IV (GET/VIN): rats received VIN (40 mg/dl)^37,38^ by oral gavage along with GET (100 mg/kg/day. i.p.)^41,42^ injection.

At the end of the experiment, rats were deeply anesthetized with thiopental (50 mg/kg)^43^, and blood was collected from a major vessel for serum separation. Subsequently, the animals were humanely sacrificed by decapitation in accordance with ethical regulations. Blood samples were spun at 4000 rpm for five minutes to separate the serum for liver and kidney function analysis. Liver and kidney tissues were obtained. The liver and kidney tissues were divided into three sections. The first section was preserved in 10% neutral buffered formaldehyde for histological analysis, while the remaining two sections were stored at -80 °C for further protein and RNA analysis.

### Liver function tests

Serum levels of alanine aminotransferase (ALT), aspartate aminotransferase (AST), and lactate dehydrogenase (LDH) were measured using reagent kits from Human Diagnostics, Germany (Cat. No. 12012 for ALT, 12021 for AST, and 12214 for LDH). All biochemical parameters were analyzed spectrophotometrically using a HumaLyzer Primus Spectrophotometer (Human Diagnostics, Germany), following the manufacturer’s instructions.

### Kidney function tests

Serum urea and creatinine levels were determined using commercial kits from Human Diagnostics, Germany (Cat. No. 10505 for urea and 10051 for creatinine), and measured spectrophotometrically using a HumaLyzer Primus Spectrophotometer (Human Diagnostics, Germany), in accordance with the manufacturer’s instructions.

Kidney Injury Molecule-1 (KIM-1) and Neutrophil Gelatinase-Associated Lipocalin (NGAL) were quantified using ELISA kits from BT Lab (Bioassay Technology Laboratory, China; Cat. No. E0549Ra for KIM-1 and E0762Ra for NGAL). ELISA measurements were performed using a Chromate SF 4300 microplate reader (Awareness Technology Inc., USA), following the protocols provided by the manufacturer.

### Levels of oxidative stress-related biomarkers

Determination of total antioxidant capacity (TAC) in serum and malondialdehyde (MDA)^44^ in liver and kidney tissues (Biodiagnositic, Giza, Egypt, Cat No # TA2513 for TAC and # MD2529 for MDA), following the instructions provided by the manufacturer.

### ELISA technique

The ELISA kits (Elabscience^®^, Houston, TX, USA) were used to quantify AMPK (Cat No # E-CL-H0257), mTOR(Cat No # E-EL-H1655), ULK1(Cat No # E-AB-67995), beclin-1(Cat No # E-EL-H0564), LC3(Cat No # E-AB-81578), and cleaved caspase-3 (Cat No # E-AB-63510) in liver and kidney tissues in compliance with the guidelines provided by the manufacturer. Absorbance was measured using an ELISA microplate reader (Chromate SF 4300, Awareness Technology Inc., USA).

### Quantitative real-time PCR (qRT-PCR) for gene expressions

The quantitative expression of Bcl-2-associated protein x *(Bax)* and B cell lymphoma-2 *(Bcl-2)* genes were assessed using qRT-PCR analysis. Liver and kidney tissues weighing 100 mg were efficiently homogenized in TRIzol reagent, using the Branson Digital homogenizer^45^. The RevertAid H Minus First Strand cDNA Synthesis Kits were used to synthesize cDNA. For the PCR reactions, the Maxima SYBR Green qRT-PCR Master Mix from (Thermo Scientific) was utilized. qRT-PCR reactions were implemented using Applied Biosystems Step One thermal cycler^46^. The comparative CT approach was employed to determine gene expression levels, with normalization to GAPDH as the reference gene^47,48^. Primer sequences of intended genes are shown in Table 1.

**Table 1:** Primer sequences of intended genes.

Gene	Forward primer	Reverse primer
*Bax*	5ʹ CACGTCTGCGGGGAGTC 3ʹ	5ʹ TGTTGTCCAGTTCATCGCCA 3ʹ
*Bcl-2*	5ʹ GGGCTACGAGTGGGATACTG 3ʹ	5ʹ GACCCCACCGAACTCAAAGA 3ʹ
*GAPDH*	5ʹ CTCTCTGCTCCTCCCTGTTC 3ʹ	5ʹ CGACATACTCAGCACCAGCA 3ʹ

### Histopathological examination

The liver and kidney tissues from all rats were carefully dissected, fixed in neutral buffered 10% formalin, dehydrated, and embedded in paraffin. These tissues were then sectioned into 6-µm slices and stained with Hematoxylin and Eosin (H&E) to assess their histological structure^49^.

#### Morphometric study

A digital camera with high resolution in color attached to a BX51 microscope (Olympus, Japan) and connected to a computer using LC Micro Application Software was used to capture the slides in the Histology and Cell Biology Department, Faculty of Medicine, Minia University, Egypt. Ten non-overlapping areas of each slide in each group were evaluated at 400× magnification. Quantitative analysis of histopathological parameters was performed using ImageJ software (National Institutes of Health, Bethesda, MD, USA; https://imagej.nih.gov/ij/).

The hepatic damage was assessed based on the extent of inflammatory and apoptotic cells, as well as the deteriorated region inside the hepatic lobules^50^.

The histopathological examination and quantification of kidney damage included the following features: “formation of medullary tubular casts, decreased RBC count in the glomerular capillaries, glomerular tuft shrinkage, Bowman’s space enlargement, congestion of the medullary blood vessels and necrosis and sloughing of epithelial cells into the proximal tubular lumen”. The sections were scored using the following system, with a range of 0 to 5: normal (score 0); up to 20% (score 1), 21–40% (score 2), 41–60% (score 3), 61–80% (score 4), more than 80% (score 5). The numerical scores for each group were recorded and their sum was used as the “total histopathological score^51^.

### Statistical analysis

ANOVA (one-way analysis of variance) was used to statistically analyze the data, followed by Tukey’s post-hoc test. Statistical calculations were performed using GraphPad Prism, version 10 for Windows (San Diego, CA, USA). Results are presented as mean ± SD, and p-values less than 0.05 were considered statistically significant.

## Results

### The impact of VIN on hepatic function tests

**Fig. 1:**
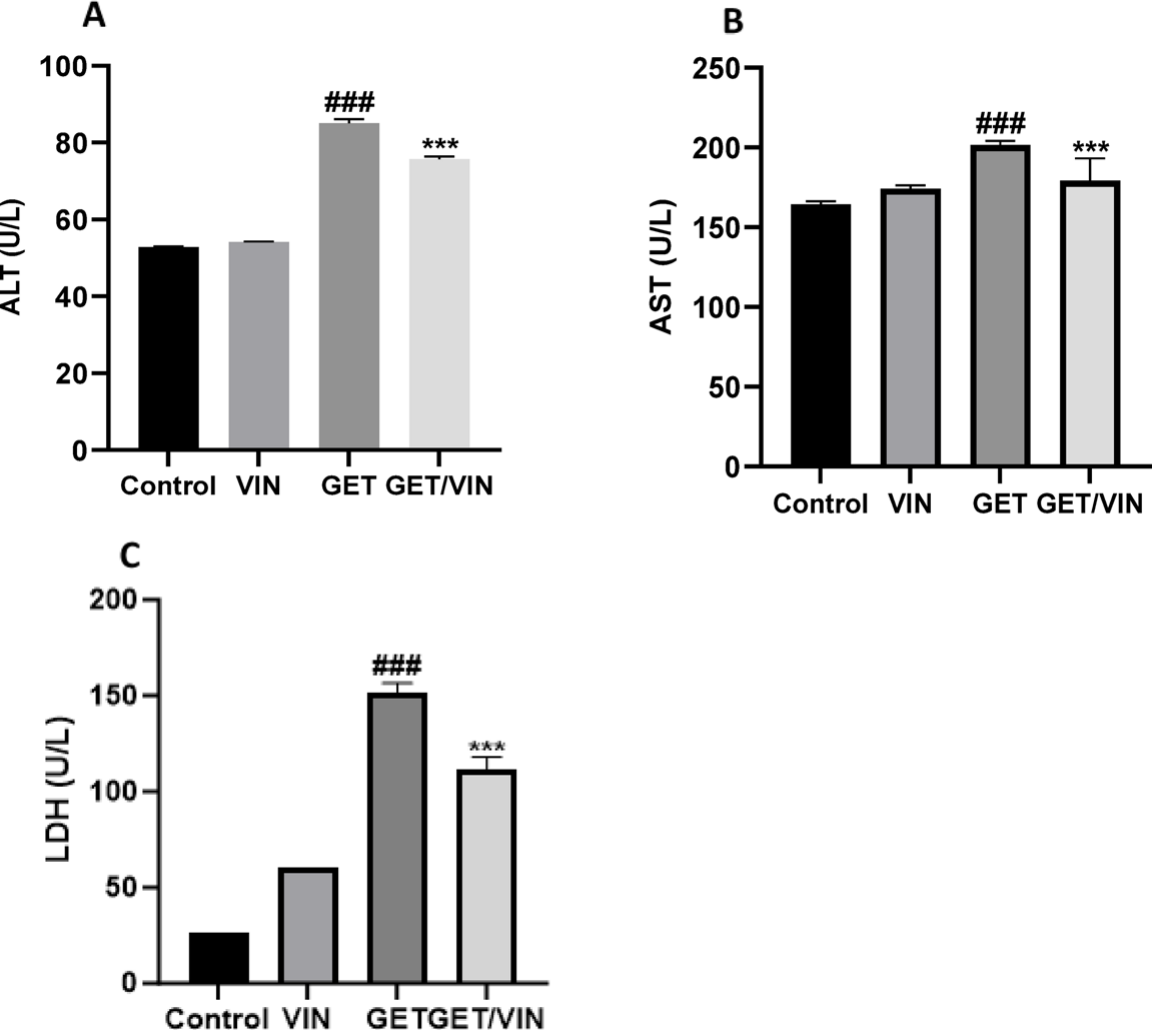
Serum levels of (**A**) ALT, (**B**) AST, and (**C**) LDH. Data are presented as mean ± SD. Statistical analysis was performed using one-way ANOVA followed by Tukey’s post-hoc test. ### *p* < 0.001, compared to the control group; *** *p* < 0.001, compared to the GET group.* ALT* alanine aminotransferase,* AST* aspartate aminotransferase,* LDH* lactate dehydrogenase.

Serum levels of ALT and AST were assessed as indicators of liver function. As shown in Fig. 1 the GET-treated group exhibited a marked elevation in ALT levels (85 U/L; *p* value < 0.0001) and AST levels (201.6 U/L; *p* value < 0.0001) compared to the control group. In contrast, the group treated with both GET and VIN (GET/VIN) displayed a significant decrease in ALT levels (75.72 U/L; *p* value < 0.0001) and AST levels (179.3 U/L; *p* value = 0.0002) compared to the GET group. Notably, no significant differences were observed between the VIN group and the control group in terms of these liver enzymes (Fig. 1A & B).

In addition, serum levels of LDH were evaluated as indicators of liver function. As demonstrated in Fig. 1, LDH levels were markedly elevated in the GET-treated group (151.7 U/L; *p* value < 0.0001) compared to the control group. However, in the GET/VIN group showed a significant reduction in LDH levels (111.2 U/L; *p* value < 0.0001) when compared to the GET group (Fig. 1C).

### The impact of VIN on kidney function tests

**Fig. 2:**
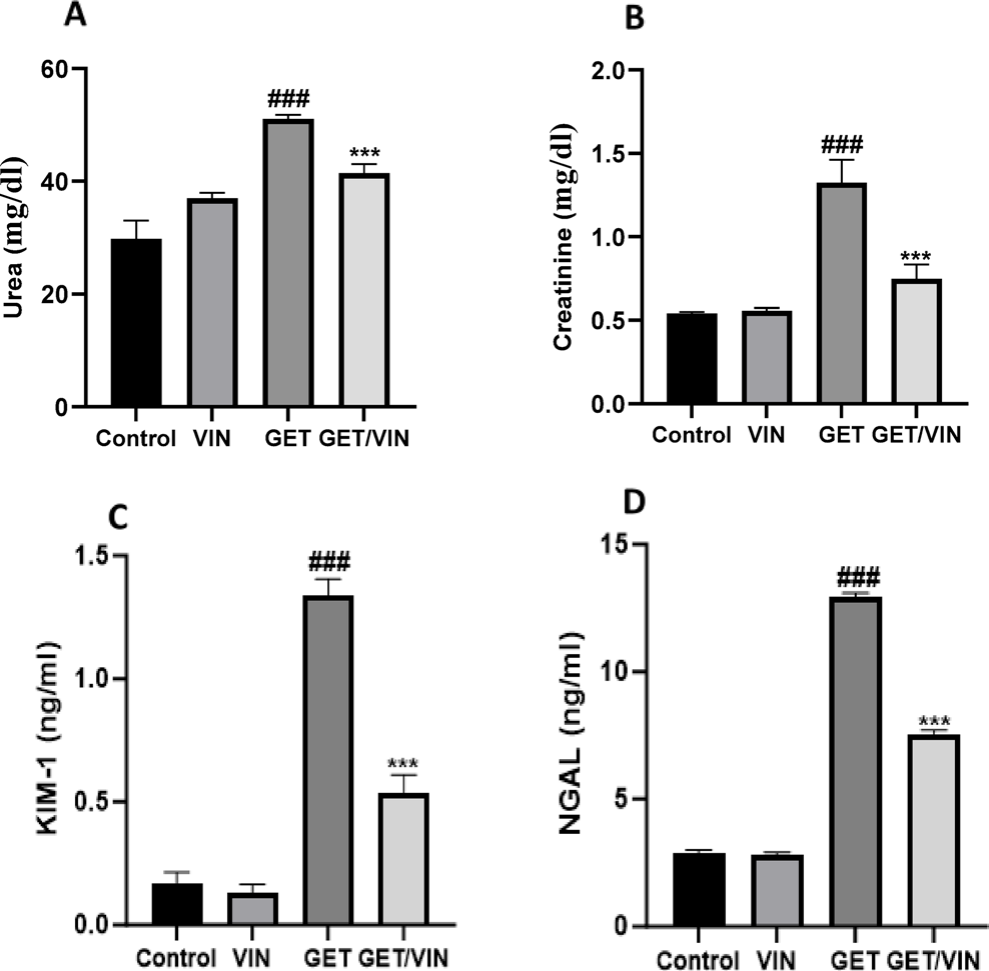
Serum levels of (**A**) urea and (**B**) creatinine, (**C**) KIM-1, and (**D**) NGAL. Data are presented as mean ± SD. Statistical analysis was performed using one-way ANOVA followed by Tukey’s post-hoc test. ### *p* < 0.001, compared to the control group; *** *p* < 0.001, compared to the GET group. *KIM-1* kidney injury molecule-1,* NGAL* neutrophil gelatinase-associated lipocalin.

To explore the impact of GET on kidney function, serum levels of urea and creatinine were assessed. The findings revealed a significant elevation in urea (51.07 mg/dl; *p* value < 0.0001) and creatinine (1.325 mg/dl; *p* value < 0.0001) levels in the GET group compared to the control group. In contrast, the group treated with GET in combination with VIN (GET/VIN) exhibited a remarkable decrease in urea (41.43 mg/dl; *p* value < 0.0001) and creatinine levels (0.7483 mg/dl; *p* value < 0.0001) in comparison to the GET group (Fig. 2 A & B).

Furthermore, KIM-1 and NGAL serum levels were assessed to indicat kidney function. The findings revealed a marked elevation in KIM-1 (1.335 ng/ml; *p* value < 0.0001) and NGAL (12.95 ng/ml; *p* value < 0.0001) levels in the GET group compared to the control group. In contrast, the GET/VIN group exhibited a remarkable decrease in KIM-1 (0.5383 ng/ml; *p* value < 0.0001) and NGAL levels (7.568 ng/ml; *p* value < 0.0001) in comparison to the GET group, highlighting the potential protective effects of VIN against GET-induced renal toxicity (Fig. 2 C & D).

### VIN reduces oxidative stress level in GET-induced liver and renal injuries

**Fig. 3:**
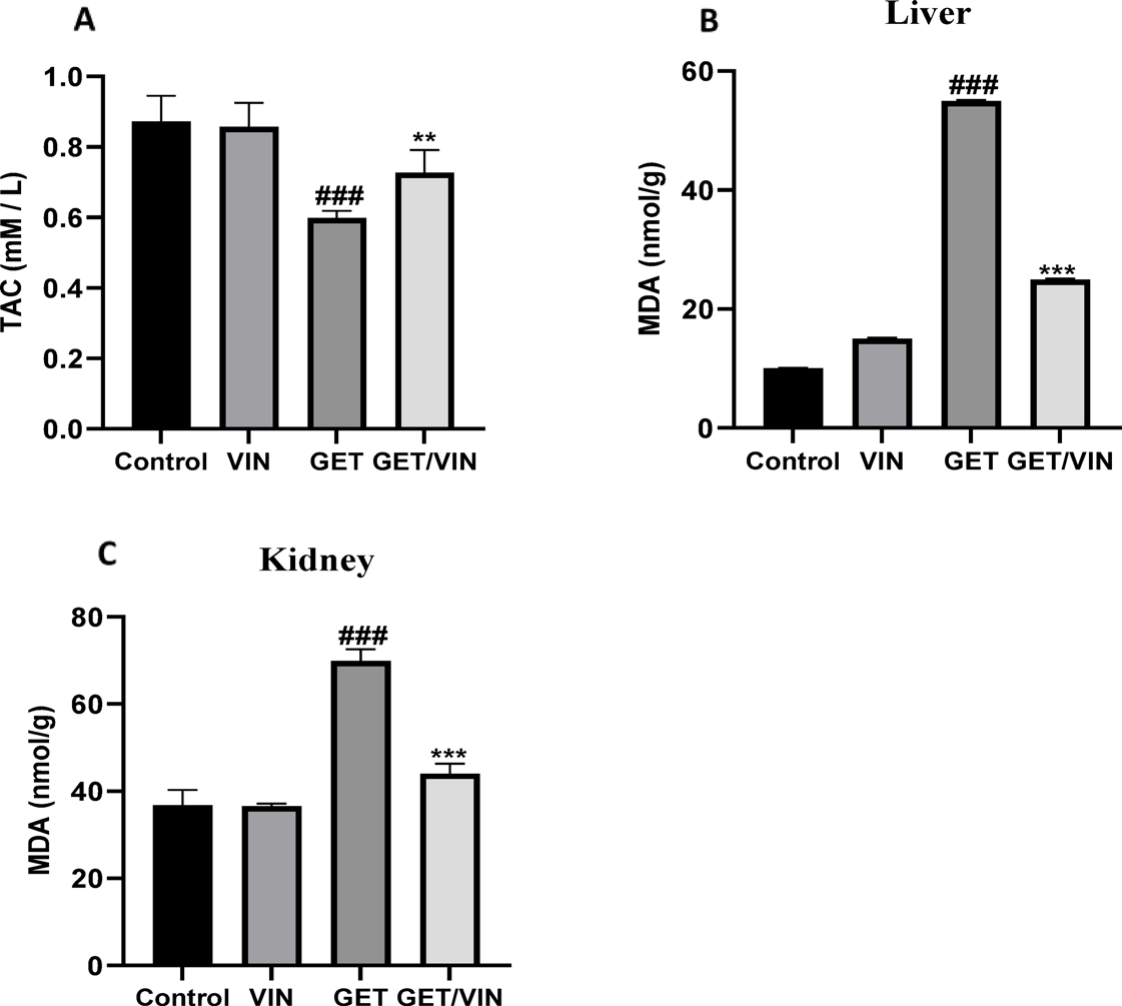
Levels of (**A**) TAC and (**B**) hepatic MDA, (**C**) renal MDA. Data are presented as mean ± SD. One-way ANOVA (*n* = 6) followed by Tukey’s post-hoc test was utilized to examine significant differences, where ###; *p* < 0.001, compared to the control group. **; *p* < 0.01, ***; *p* < 0.001 compared to the GET group. *TAC* total antioxidant capacity,* MDA* malondialdehyde.

Figure 3 depicts the concentrations of TAC in serum of different groups. Serum TAC level in the GET group were markedly (0.5985 mM/L; *p* value < 0.0001) decreased, with respect to control group. Nevertheless, GET/VIN group (0.7272 mM/L; *p* value = 0.0073) exhibited a significant reversal of this alteration compared to the GET group (Fig. 3A).

Figure 3 also shows that MDA was considerably higher in the GET groups in liver (54.97 nmol/gm; *p* value < 0.0001) and kidney tissues (69.93 nmol/gm; *p* value < 0.0001), compared to control group. Conversely, MDA levels were considerably dropped in GET/VIN group in liver (24.98 nmol/gm; *p* value < 0.0001) and kidney tissues (44.00 nmol/gm; *p* value < 0.0001), in contrast to GET group (Fig. 3B & C).

### Effect of VIN on AMPK, beclin-1, and LC3 in liver and kidney tissues

In this study, we investigated whether VIN’s therapeutic potential was linked to autophagy. Therefore, our focus was on the expression of AMPK, mTOR, LC3, and beclin-1 proteins as a reliable indicator of the autophagic process^22^. The hepatic levels of AMPK, beclin-1, and LC3 proteins were measured using ELISA.

**Fig. 4:**
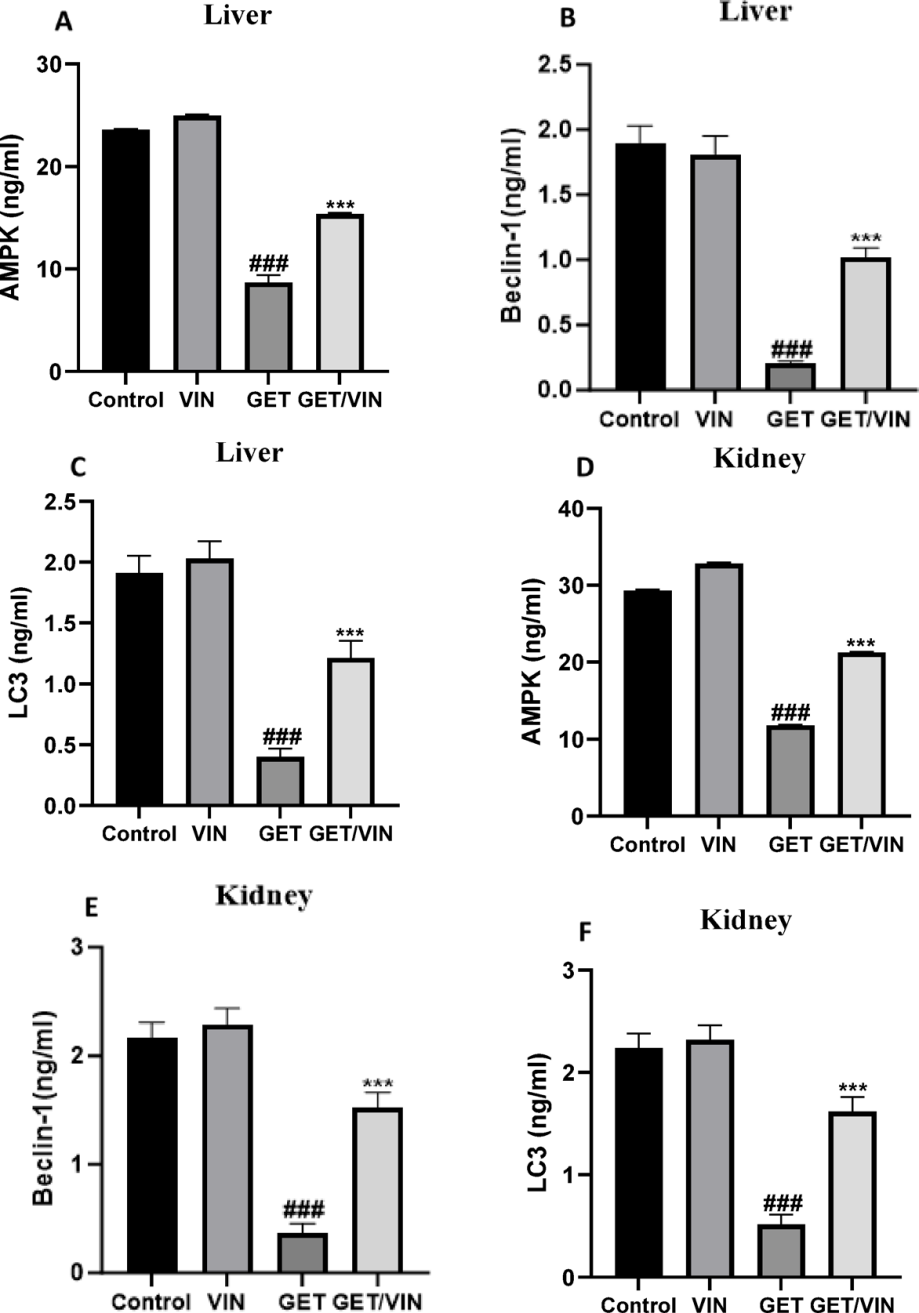
Levels of (**A**) hepatic AMPK, (**B**) hepatic beclin-1, (**C**) hepatic LC3, (**D**) renal AMPK, (**E**) renal beclin-1, (**F**) renal LC3. Each bar represents the mean ± SD. Significant differences (*n* = 6) were examined using a one-way ANOVA followed by Tukey’s post hoc test, where ###; *p* < 0.001, compared to control group, ***; *p* < 0.001, compared to the GET group.* AMPK *AMP-activated protein kinase,* LC3* microtubule-associated protein light chain 3.

In the GET group, the level of AMPK significantly decreased (8.697 ng/ml, *p* value < 0.0001) compared to the control group. Nevertheless, in the GET/VIN group, AMPK level significantly increased to 15.35 ng/ml, *(p* value < 0.0001) compared to the GET group (Fig. 4 A).

Similarly, beclin-1 levels in the GET group were significantly reduced to 0.2008 ng/ml, (*p* value < 0.0001) compared to the control group. However, in the GET/VIN group, beclin-1 levels markedly increased to 1.022 ng/ml, (*p* value < 0.0001) compared to the GET group (Fig. 4B).

Likewise, LC3 levels in the GET group were significantly decreased compared to the control group (0.4017 ng/ml, *p* value < 0.0001). However, in the GET/VIN group, LC3 levels significantly increased compared to the GET group (1.212 ng/ml, *p* value < 0.0001) (Fig. 4C).

Additionally, the kidney levels of AMPK, beclin-1, and LC3 proteins were also detected using ELISA. In the GET group, the level of AMPK significantly decreased to 11.82 ng/ml, (*p* value < 0.0001) compared to the control group. However, in the GET/VIN group, AMPK level significantly increased (21.26 ng/ml, *p* value < 0.0001) compared to the GET group (Fig. 4D).

Beclin-1 levels in kidneys of GET group were significantly reduced compared to the control group (0.3700 ng/ml, *p* value < 0.0001). In contrast, beclin-1 levels markedly increased (1.522 ng/ml, *p* value < 0.0001) in the GET/VIN group compared to the GET group (Fig. 4E).

Renal LC3 levels were significantly decreased (0.5150 ng/ml, *p* value < 0.0001) in the GET group compared to the control group. In addition, LC3 levels significantly increased (1.618 ng/ml, *p* value < 0.0001) in the GET/VIN group compared to the GET group (Fig. 4F).

### Effect of VIN on hepatic and renal mTOR

**Fig. 5:**
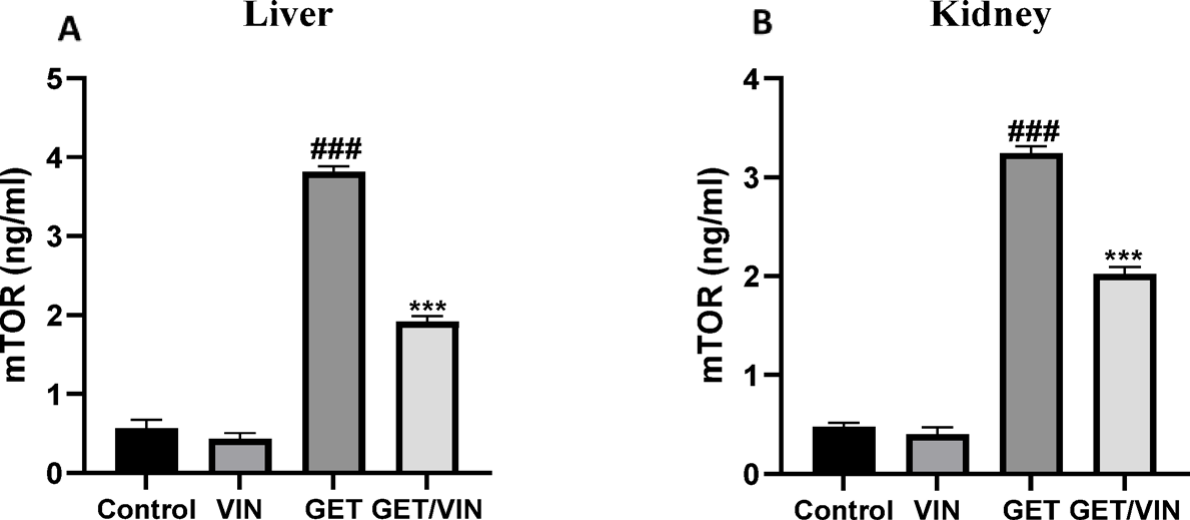
Levels of (**A**) hepatic mTOR and (**B**) renal mTOR. Data are expressed as mean ± SD (*n* = 6). Statistical significance was determined using one-way ANOVA followed by Tukey’s post hoc test, where ###; *p* < 0.001, in contrast to control group, ***; *p* < 0.001, by contrast with GET group. *mTOR* mammalian target of rapamycin.

Hepatic and renal mTOR levels were determined using ELISA technique. The results showed that the levels of hepatic and renal mTOR were markedly elevated (hepatic mTOR: 3.815 ng/ml, renal mTOR: 3.243 ng/ml), (*p* value < 0.0001) in GET group with respect to control group. Conversely, the GET/VIN group showed a significant reduction in mTOR levels (hepatic mTOR; 1.919 ng/ml, renal mTOR; 2.020 ng/ml), (*p* value < 0.0001) in comparison to the GET group (Fig. 5).

### Effect of VIN on hepatic and renal cleaved caspase-3

**Fig. 6:**
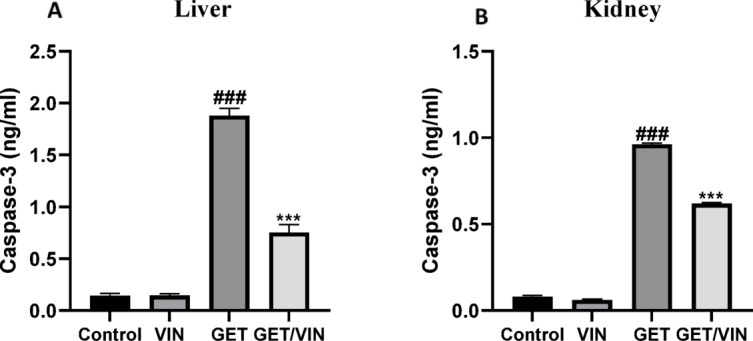
Levels of (**A**) hepatic cleaved caspase-3 and (**B**) renal cleaved caspase-3. Each bar represents the mean ± SD. Significant differences (*n* = 6) were examined using a one-way ANOVA followed by a Tukey post-hoc test, where ###; *p* < 0.001, in contrast to control group, ***; *p* < 0.001, by contrast with GET group.

Cleaved caspase-3 level was investigated using ELISA in both liver and kidney tissues. It has been shown that hepatic cleaved caspase-3 was significantly raised in the GET group with respect to control group **(**1.880 ng/ml), (*p* value < 0.0001). Conversely, in the GET/VIN group it led to a significant decrease in this level to 0.7533 ng/ml, (*p* value < 0.0001) in comparison to GET group (Fig. 6A).

Regarding renal tissues, cleaved caspase-3 levels were markedly elevated in GET group with respect to control group (0.9613 ng/ml), (*p* value < 0.0001). Conversely, in the GET/VIN group it led to a significant decrease in levels (0.6190 ng/ml), (*p* value < 0.0001) in comparison to GET group (Fig. 6B).

### Effect of VIN on mRNA expression of *Bax* and *Bcl-2* in liver and kidney tissues

**Fig. 7:**
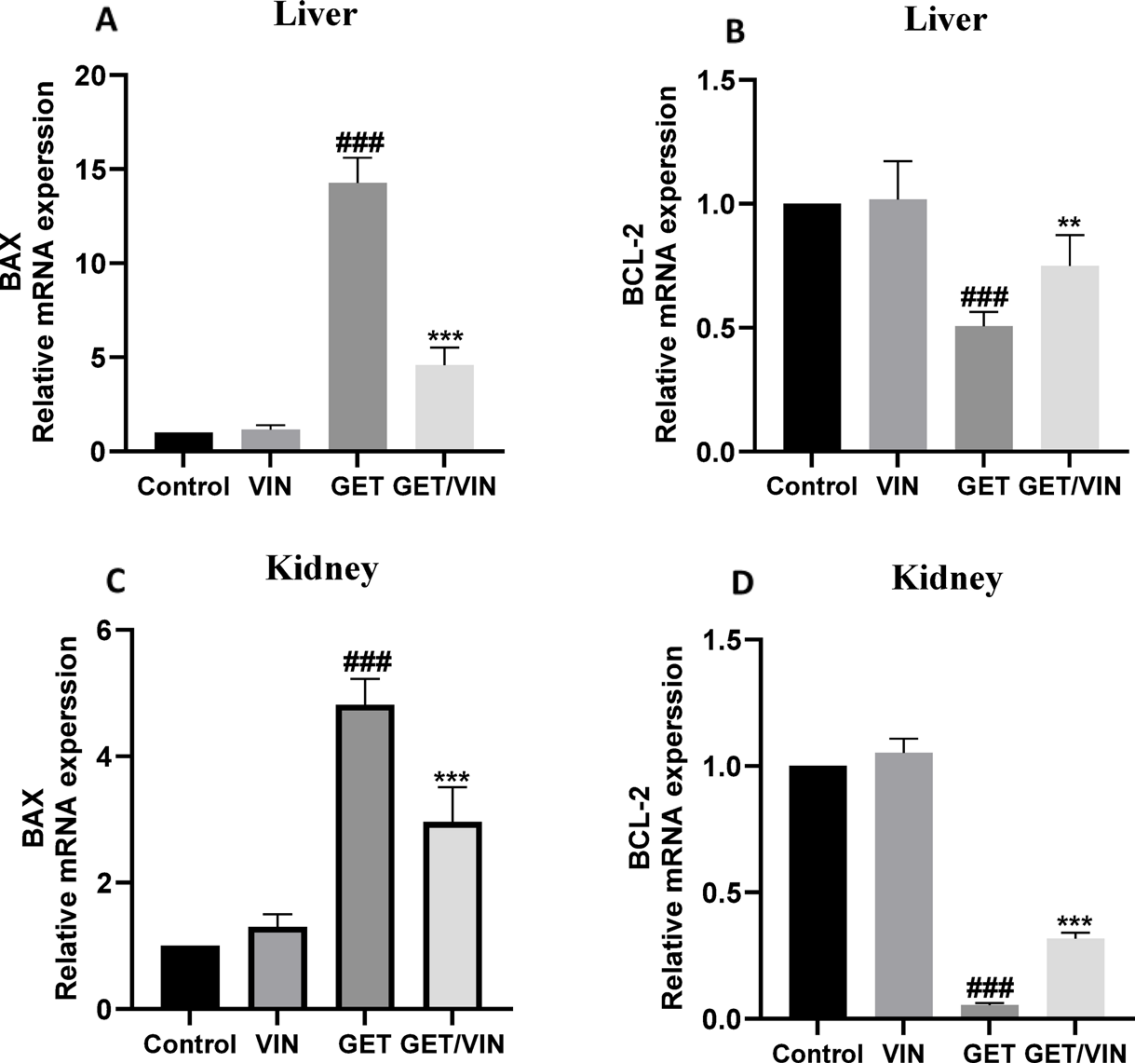
The impact of VIN on the expression of (**A**) *Bax* and (**B**) *Bcl-2* in liver. (**C**) *Bax* and (**D**) *Bcl-2* in kidney. Bars represent mean ± SD (*n* = 6). Gene expression was quantified using real-time PCR and presented as fold change relative to the control group. GAPDH was used as the housekeeping gene. Statistical significance was determined using one-way ANOVA followed by Tukey’s post hoc test, where ###; *p* < 0.001, compared to control group. ***; *p* < 0.001, compared to the GET group. *Bax* Bcl-2-associated protein x,* Bcl-2* B cell lymphoma-2.

To assess *Bax and Bcl-2* mRNA expression, real-time PCR was used. Proteins belonging to the *Bcl-2* family are necessary for regulating apoptosis. Two important members of this family are *Bax and Bcl-2*. In liver tissue, the GET group demonstrated a substantial increase in *Bax* compared to the control group (14.27-fold; *p* value < 0.0001). On the contrary, a significant decrease was noticed in *Bax* in GET/VIN group in comparison with the GET group (4.587-fold; *p* value < 0.0001). However, the expression of *Bcl-2* decreased in the GET group (0.5064-fold; *p* value < 0.0001) and was recovered in GET/VIN group compared to the GET group (0.7496-fold; *p* value = 0.0029), as illustrated in Fig. 7 A, B.

In kidney tissue, the GET group demonstrated a substantial increase in *Bax* compared to the control group (4.818-fold; *p* value < 0.0001). On the contrary, a significant decrease was noticed in *Bax* in GET/VIN group in comparison with the GET group (2.965-fold; *p* value < 0.0001). However, the expression of *Bcl-2* decreased in the GET group (0.05442-fold; *p* value < 0.0001) and was significantly recovered in GET/VIN groups compared to the GET group (0.3180-fold; *p* value < 0.0001), as illustrated in Fig. 7 C, D.

### Histopathological results

**Fig. 8:**
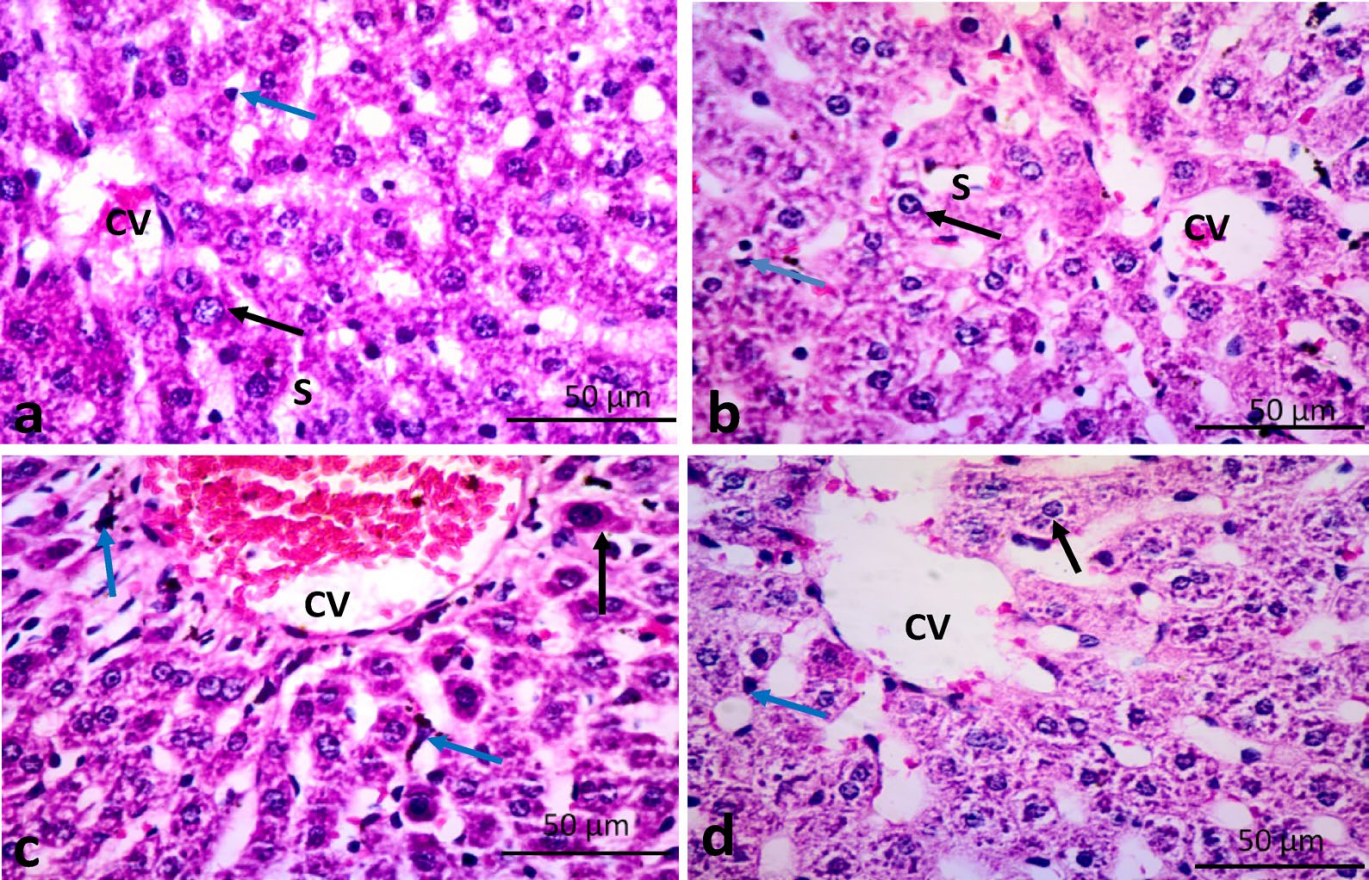
Photomicrographs of rat liver tissue of: (**a**,**b**) control group and VIN group showing normal liver architecture. The liver tissue is formed of hepatocytes (black arrows) with vesicular nuclei radiating from central veins (CV) and separated by blood sinusoids (S). Notice the Kupffer cells (blue arrows) are observed within the sinusoids. (**c**) GET group shows a dilated congested central vein (CV), darkly stained hepatocytes, and large sized Kupffer cells containing dark granules. (**d**) GET/VIN group demonstrates an apparently normal liver tissue. Hematoxylin and Eosin stain, original magnification X 400 (H&E X 400, scale bar = 50 μm).

**Fig. 9:**
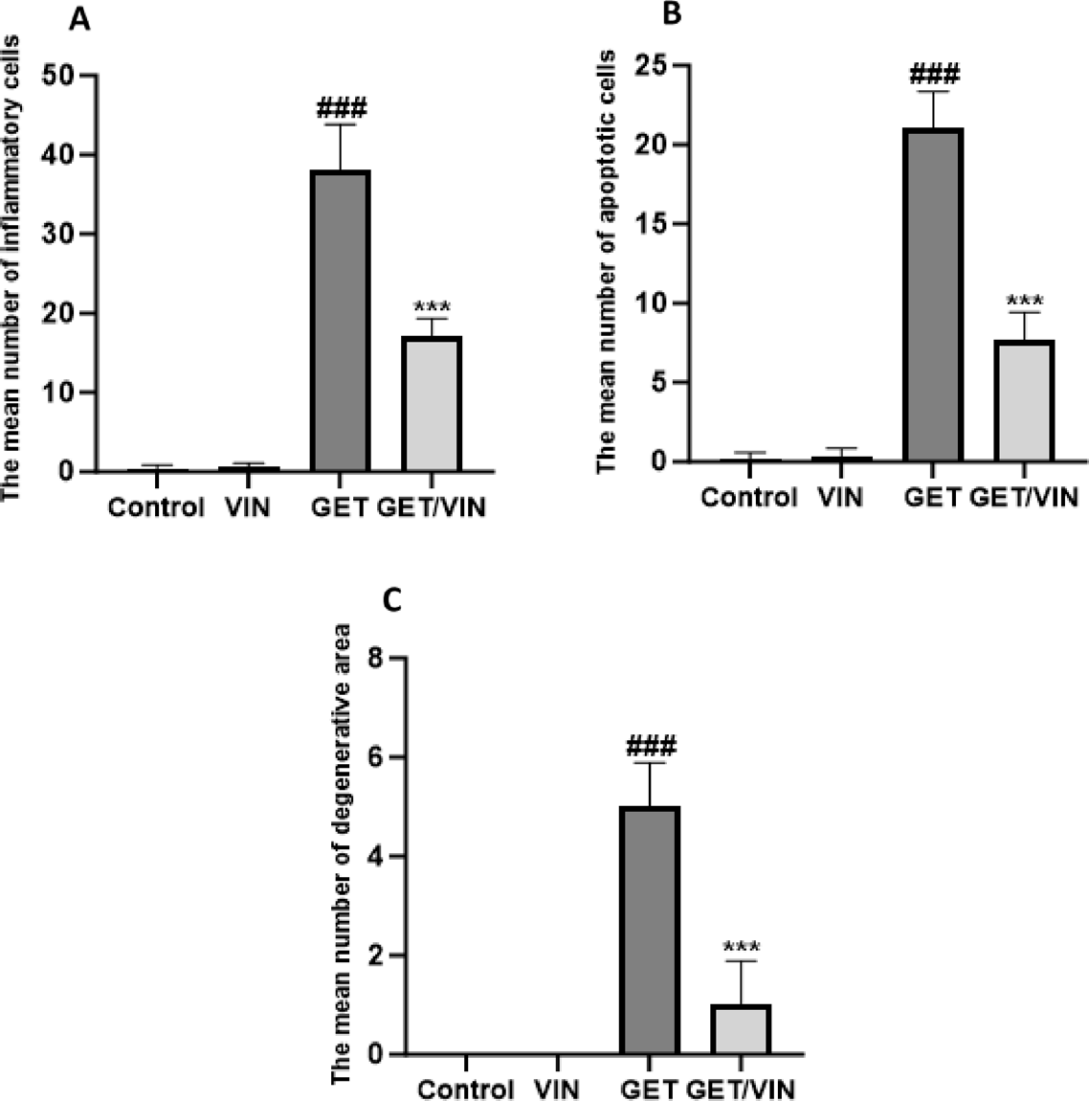
Morphometric analysis based on semi-quantitative scoring of (**A**) the mean number of inflammatory cells, (**B**) the mean number of apoptotic cells, and (**C**) the mean number of degenerative areas. Data are expressed as the mean number of cells per high-power field (HPF) at 400x magnification ± SD (*n* = 6). Statistical analysis was performed using one-way ANOVA followed by Tukey’s post hoc test. ### *p* < 0.001 vs. control group; *** *p* < 0.001 vs. GET group.

Liver sections from the control and VIN groups exhibited normal histological architecture. The liver tissue was formed of cords of hepatocytes with vesicular nuclei radiating from central veins. The hepatocytes were separated by blood sinusoids. Kupffer cells, characterized by their irregular shapes, were observed within these sinusoids. In contrast, the GET group showed notable pathological changes, including dilated and congested central veins, darkly stained hepatocytes, and enlarged Kupffer cells containing dark granules. Meanwhile, liver sections from the GET/VIN group appeared to exhibit nearly normal histological features, closely resembling the control structure (Figs. 8 and 9).

Renal sections from control and VIN groups exhibited normal kidney structure. The kidney was formed of renal corpuscles (formed of renal glomeruli and Bowman’s capsules), proximal (with narrow lumen), distal (with dilated lumen) convoluted tubules with vesicular nuclei. However, the GET group showed distorted shrunken renal corpuscles with less cellularity and dilated Bowman’s spaces. Tubular cells with darkly stained cytoplasm and nuclei were also observed. Intra luminal hyaline and inflammatory cell infiltration were also a clear finding. While in the GET/VIN group displayed renal tissue nearly approach normal structure except for glomerular congested blood capillaries (. 10).

**Fig. 10:**
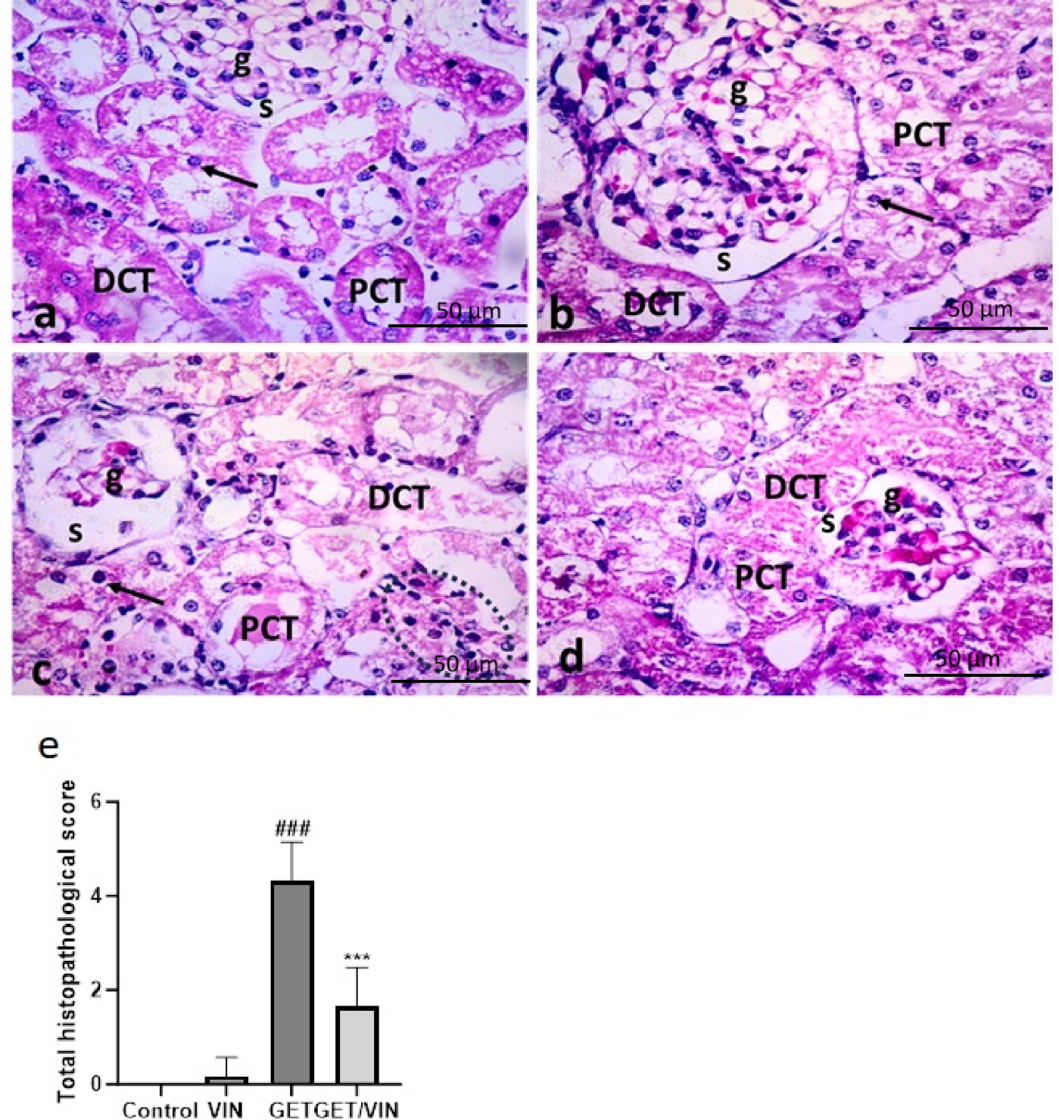
Photomicrographs of rat renal tissue of: (**a**,**b**) control group and VIN group showing normal kidney structure. The renal cortex is formed of renal glomerulus (g) (surrounding by Bowman’s space (s), proximal (PCT) & distal (DCT) convoluted tubules with vesicular nuclei. (**c**) GET group displays a distorted, shrunken renal glomerulus (g) with dilated Bowman’s space (s), darkly stained cells (arrows) of proximal (PCT) & distal (DCT) convoluted tubules, intra luminal hyaline, and inflammatory cell infiltration. (**d**) GET/VIN group reveals kidney tissue nearly approach normal structure except for glomerular congested blood capillaries. (H&E X 400, scale bar = 50 μm). (**e**) Analysis of Semi-quantitative scoring. Data are presented as mean ± SD, (*n* = 6). Statistical analysis was performed using one-way ANOVA followed by Tukey’s post hoc test. Where ###; *p* < 0.001, compared to the control group, ***; *p* < 0.001, compared to the GET group. The analysis was performed per high-power field (HPF).

## Discussion

Despite its affordability, availability, and specificity^52^, GET is frequently associated with severe adverse effects, particularly nephrotoxicity and hepatotoxicity^4,8^. This may restrict the clinical use of aminoglycosides, as deteriorations in liver and kidney function are commonly observed within a week of administration^53,54^. Several strategies employing synthetic compounds have been explored to counteract the hepatic and renal toxicity of GET^13,29^. Nonetheless, limited attention has been given to the potential of naturally occurring substances with potent antioxidant properties to mitigate hepatotoxic and nephrotoxic harm induced by GET. In this context, we have investigated the possible safeguard efficacy of VIN against GET-triggered hepatorenal toxicity. The results of our research revealed, for the first time, that VIN exerted ameliorating effects on GET toxicity by alleviating oxidative stress, preventing apoptosis through attenuating the Bax/Bcl-2 signaling pathway, and stimulating the AMPK/mTOR autophagy signaling pathway.

The findings of this study reveal a substantial elevation in serum creatinine and urea levels following GET administration. These results are in contrast with those of Guidet and Shah’s research^55^, who reported no changes in these parameters after administering GET at 100 mg/kg/day for five consecutive days. Nevertheless, our findings are consistent with previous studies^56^. This discrepancy may be attributed to the difference in treatment duration with GET. In our investigation, rats received GET at 100 mg/kg/day for seven days, compared to five days in the study by Guidet and Shah^55^. Our results support the idea that changes in glomerular filtration rate, serum creatinine, BUN as well as the early biomarkers of nephrotoxicity such as KIM-1 and NGAL, typically appear after 7 days of GET treatment^57,58^. Consistent with the histopathological analyses, the results demonstrated notable splitting, shrinking, and increased cell density in the glomerular tufts, along with pronounced hypercellularity in the capillary tufts. Furthermore, significant vacuolation and necrosis were observed in the epithelial linings of both the proximal and distal convoluted tubules. However, incamine exhibited a protective effect on the kidneys against GET-triggered injury, as evidenced by the improved levels of the examined biomarkers and the observed histological modifications.

In our investigation, the hepatic injury was induced in rats through intraperitoneal injection of GET, as evidenced by elevated serum levels of ALT, AST and LDH, reflecting impaired liver function. These findings align with previous studies^9,59,60^. Notably, co-administration of GET and VIN significantly improved liver function, as evidenced by reduced ALT, AST and LDH levels. This suggests that VIN may protect the integrity of the hepatocyte membrane, thereby preventing the GET-induced leakage of liver enzymes into the bloodstream. Histopathological assessments further supported our findings, revealing that GET led to ballooning, necrosis, and inflammation within hepatocytes, whereas treatment with VIN ameliorated these GET -induced changes. These promising results pave the way for deeper exploration of VIN’s hepatoprotective properties.

Given the central roles of oxidative stress and inflammation in GET-induced hepatic and renal toxicity, we hypothesized that the protective effects of VIN were associated with the attenuation of these mechanisms. GET administration has been associated with mitochondrial dysfunction, increased production of ROS, and other adverse effects. ROS are potent oxidizing agents that can damage lipids, proteins, and DNA through oxidative mechanisms^6^. Serum TAC serves as a valuable indicator of the body’s overall redox status and oxidative balance. Serum TAC measurement may be used to diagnose oxidative status-affecting diseases. Rather than calculating the simple sum of all detectable antioxidants, the TAC measurement takes into account the combined effect of all antioxidants found in serum and body fluids^61,62^. MDA, a highly reactive aldehyde, is produced during the polyunsaturated fatty acid lipid peroxidation. This compound serves as a key biomarker for oxidative stress^63,64^. Consistent with earlier findings, our study revealed a substantial decrease in TAC and an increase in MDA concentrations in the GET group in the liver and kidney, highlighting the role of oxidative stress in GET toxicity^2,8^. However, in the GET/VIN group, these alterations were counteracted, as reflected by increased TAC and decreased MDA levels. These results suggest that the protective effects of VIN are mediated by its antioxidant potential. These findings are verified by earlier research that had observed a significant reduction of oxidative stress by VIN in different rat models^37^.

Autophagy is pivotal for several cellular processes encompassing the clearance of damaged cellular proteins and organelles, enhancing cellular energy production, and alleviation of endoplasmic reticulum stress. Hence, autophagy plays a central role in maintaining cellular homeostasis and viability. In the realm of kidney function preservation, basal autophagy emerges as indispensable for the survival of glomerular mesangial cells, proximal tubular epithelial cells, glomerular endothelial cells, and podocytes^65^. Moreover, recent findings underscore the critical role of autophagic activity in liver diseases such as NAFLD and AFLD, as well as in exacerbating hepatotoxicity induced by viral infections, aflatoxins, or heavy metals^66^. Extensive reviews have emphasized the regulatory role of autophagy in liver diseases^67,68^. Within the autophagic framework, beclin-1 plays a central role in orchestrating autophagosome development throughout the sequestration phase, thereby stimulating autophagic activation^16^. Our results illustrated that GET led to a notable impairment in autophagy, as evidenced by marked downregulation of beclin-1 expression in hepatic and renal tissues. This observation underscores a compelling link between the derangement of autophagic processes and the pathogenesis of GET-induced hepatorenal injury^9,69,70^. In our experimental model, the disruption of autophagy observed in the GET group is consistent with previous papers about GET-induced toxicity in rat models^29^. Compelling evidence underscores the potential of drug candidates aimed at enhancing autophagy in hepatic and renal cells to alleviate hepatic and renal impairments^9,29,70,71^. Notably, VIN, as demonstrated in this study, exhibits the capacity to activate the autophagic machinery, as evidenced by the upregulation of beclin-1 expression.

In a bid to further validate autophagy induction, we assessed LC3-II levels, a key molecular marker of autophagic activity, as the LC3-I to LC3-II conversion is a hallmark of autophagy^72^. Our findings revealed a significant decrease in LC3-II protein expression in the GET group relative to the control group, a change that was reversed following VIN intervention.

The current findings have brought to light a pronounced suppression of the AMPK/mTOR pathway, a critical regulator of autophagy, following GET administration. This suppression was evidenced by elevated p-mTOR expression. These findings are consistent with earlier studies reporting AMPK/mTOR pathway inhibition in lipopolysaccharide (LPS)-induced liver inflammation^73^, diabetic nephropathy, and cisplatin-induced renal injury models^72,74^. This observation aligns with the recognition that the energy-sensing AMPK plays a pivotal role in sustaining cellular energy levels by kickstarting autophagic processes through the regulation of the p-mTOR/t-mTOR ratio^18^. mTOR, a key upstream regulator of autophagy, inhibits downstream autophagy signaling proteins when hyperactivated^75,76^. Furthermore, elevated mTOR activity in podocytes can impair autophagy control, leading to autophagic cell death, which is a critical factor in the progression of diabetic nephropathy^77,78^. The involvement of mTOR activation has also been demonstrated in liver cell injury induced by LPS^73^. The current study unveiled that VIN effectively restored AMPK/mTOR signaling in hepatic and renal tissues, as evidenced by the increased p-AMPK levels and decreased p-mTOR levels, thereby fostering autophagy induction.

Impaired autophagy leads to the accumulation of ROS and damaged mitochondria within cells, setting off apoptotic pathways that culminate in cell death^79^. This apoptotic cascade involves the activation of caspase-3^80,81^, a process known to hinder autophagy initiation through the caspase-3-mediated degradation of beclin-1^82^. In cases of GET induced toxicity, apoptosis is predominantly mediated via the mitochondrial pathway^83^. The delicate balance between the anti-apoptotic Bcl-2 and pro-apoptotic Bax proteins is essential for mitochondrial membrane maintenance^84^. This align with previous findings that highlighted the established link between oxidative stress, apoptotic cell death induction, and GET-induced toxicity^85,86^. In support of these findings, the current study reveals that GET triggers pro-apoptotic processes in the liver and kidney, as evidenced by elevated Bax gene expression and reduced Bcl-2 gene expression, which ultimately leads to caspase-3 activation, as shown by the higher levels of cleaved caspase-3 in rats treated with GET. VIN effectively inhibited these pro-apoptotic responses, as indicated by elevated Bcl-2 expression, reduced Bax expression, and decreased cleaved caspase-3 levels. These beneficial events are closely related to the survival of renal and liver tissues as well as the strengthening of autophagy processes^87,88^. Our findings are in agreement with earlier research that highlight how VIN simultaneously modulates the apoptotic response and activates autophagy^89^.

## Conclusions

Our findings suggest that VIN may attenuate hepatotoxicity and nephrotoxicity associated with GET through multiple mechanisms. These positive outcomes are the result of VIN’s diverse actions, which include anti-apoptotic, and antioxidant processes through the activation of the beclin-1/AMPK/mTOR pathways. These findings support the potential use of VIN as an adjunct therapeutic strategy for managing GET-induced hepatorenal dysfunction. While the results of the current study showed that VIN may serve as a promising prophylactic candidate for gentamicin-induced hepato- and nephrotoxicity, further dose-response analyses and clinical studies are required to validate the results.

## Data Availability

All data are included in the manuscript.
